# Cytokines, hepatic cell profiling and cell interactions during bone marrow cell therapy for liver fibrosis in cholestatic mice

**DOI:** 10.1371/journal.pone.0187970

**Published:** 2017-11-27

**Authors:** Daphne Pinheiro, Luana Leirós, Juliana Barbosa Torreão Dáu, Ana Carolina Stumbo, Alessandra Alves Thole, Erika Afonso Costa Cortez, Carlos Alberto Mandarim-de-Lacerda, Lais de Carvalho, Simone Nunes de Carvalho

**Affiliations:** 1 Laboratory of Stem Cell Research, Department of Histology and Embryology, Institute of Biology Roberto Alcântara Gomes, State University of Rio de Janeiro, Rio de Janeiro, Brazil; 2 Laboratory of Morphometry and Cardiovascular Morphology, Anatomy Department. Institute of Biology Roberto Alcântara Gomes, State University of Rio de Janeiro, Rio de Janeiro, Brazil; Centro Cardiologico Monzino, ITALY

## Abstract

Bone marrow cells (BMC) migrate to the injured liver after transplantation, contributing to regeneration through multiple pathways, but mechanisms involved are unclear. This work aimed to study BMC migration, characterize cytokine profile, cell populations and proliferation in mice with liver fibrosis transplanted with GFP^+^ BMC. Confocal microscopy analysis showed GFP^+^ BMC near regions expressing HGF and SDF-1 in the fibrotic liver. Impaired liver cell proliferation in fibrotic groups was restored after BMC transplantation. Regarding total cell populations, there was a significant reduction in CD68^+^ cells and increased Ly6G^+^ cells in transplanted fibrotic group. BMC contributed to the total populations of CD144, CD11b and Ly6G cells in the fibrotic liver, related to an increment of anti-fibrotic cytokines (IL-10, IL-13, IFN-γ and HGF) and reduction of pro-inflammatory cytokines (IL-17A and IL-6). Therefore, HGF and SDF-1 may represent important chemoattractants for transplanted BMC in the injured liver, where these cells can give rise to populations of extrahepatic macrophages, neutrophils and endothelial progenitor cells that can interact synergistically with other liver cells towards the modulation of an anti-fibrotic cytokine profile promoting the onset of liver regeneration.

## Introduction

Liver fibrosis is characterized by parenchymal chronic injury followed by extracellular matrix (ECM) accumulation. Cirrhosis is the most advanced stage of liver fibrosis, leading to hepatic failure and with high rates of morbidity and mortality worldwide. Liver transplantation is the only effective therapy for cirrhosis currently, and insufficient compatible donors prompt the search for new therapies [1,2,3]. Evidence show that bone marrow cells (BMC) can restore liver function in chronic lesions, acting in a paracrine manner. Two stem cell populations are found in BMC fraction: hematopoietic stem cells (HSC), which give rise to all blood cells, and mesenchymal stem cells (MSC), which interact with HSC in the specialized hematopoietic niche in the bone marrow, and are recognized for its immunomodulatory effects and plasticity to differentiate mainly in chondrocytes, osteocytes, adipocytes, fibroblasts and pericytes. Several studies have shown that BMC transplantation is associated with liver regeneration in clinical trials and animal models [4,5].

Bile duct ligation (BDL) is a well-known experimental model to induce liver fibrosis in rodents. This model consists in bile flow interruption (cholestasis), leading to morphological and pathophysiological changes similar to those observed in biliary cirrhosis. During cholestasis, bile acids remain retained in liver parenchyma causing damage in hepatocytes and triggering inflammatory mechanisms [6,7].

Damaged hepatocytes and cholangiocytes release inflammatory mediators that recruit local leukocytes to the site of injury. These leukocytes amplify inflammation through production of pro-inflammatory cytokines such as interleukin-6 (IL-6), interleukin 1-beta (IL-1β), tumor necrosis factor-alpha (TNF-α), followed by recruitment of T cells [8,9]. Two cell types are responsible for ECM deposition in cholestatic disease, acting as fibrogenic cells in the liver: portal myofibroblasts which are fibroblasts transdifferentiated by TGF-β (transforming growth factor beta), and subendothelial hepatic stellate cells (HS), that assume a myofibroblast-like phenotype when activated by TGF-β. Kupffer cells are intra-hepatic macrophages that once activated play a role as positive modulators of liver fibrosis and stimulate fibrogenic cells activation [10,11].

Different cell phenotypes have shown to accomplish anti-fibrotic effects in the injured liver in experimental models. The macrophage subpopulation classified as M2 has an anti-inflammatory nature displaying a Th2 cytokine profile. These macrophages could negatively modulate fibrosis releasing cytokine IL-10. Interestingly, neutrophils and Kupffer cells under certain stimulus and conditions within damaged hepatic microenvironment can produce specific types of MMP (metalloproteinases), which are pivotal enzymes to stop ECM deposition and allow tissue remodeling [12,13]. It is known that some MMPs are positively modulated after cell therapy in the cholestatic liver, and that extra-hepatic macrophages are key cells in their production, suggesting an increment given by transplanted BMC to this cell population [14]. BMC are capable of originating other important cell types, however their participation in fibrosis regression after cell therapy is unclear, as well as the main cytokines produced by the newly formed cells from transplanted BMC. The consequences and effects of these emerging cells interacting with injured parenchymal cells in the cholestatic liver are also widely unknown.

Furthermore, the mechanisms involved in BMC migration and establishment within liver tissue are still under investigation. Some molecules have been described as chemotactic factors in fibrosis followed by carbon tetrachloride-induced hepatotoxicity, such a SDF-1 (stromal cell-derived factor 1) and HGF (hepatocyte growth factor) [15, 16], but there is no data concerning the role of these molecules in cholestatic disease models. Therefore, the current work aimed to investigate factors and cell phenotypes derived from transplanted BMC that contribute to liver regeneration in cholestatic disease, taking a glance in the causal modulation over other hepatic cells. We also searched for molecules that could guide the migration of BMC cells to the liver with fibrosis induced by bile duct ligation in mice.

## Materials and methods

### Experimental groups

Male (2 months old), wild type C57BL/6 mice were used in this study. All animal proceedings were previously submitted to analysis and fully approved by the State University of Rio de Janeiro’s Animal Care and Use Committee (CEUA 032/2012). The animals were divided into five groups (n = 7): normal (healthy) animals, normal animals that received BMC and were euthanized after 7 days (N+BMC7d), animals with 7 days of fibrosis (F7d), animals with 14 days of fibrosis (F14d) and a group with 7 days of fibrosis that received BMC transplantation and was euthanized after 7 days (F7d+BMC7d).

### Bile duct ligation

C57BL/6 mice were anesthetized with inhaled isoflurane. After a midline laparotomy (2 cm), the common bile duct was exposed and ligated in each of its extremities with non-absorbable 6–0 suture. The bile duct was cut between ligations and the abdominal layers were sutured with 6–0 Vicryl (Ethicon, Johnson & Johnson). Animals were immediately transferred to cages with food and water *ad libitum* and monitored in the first 24 hours after surgery proceedings. Histologic and hepatic enzyme analysis confirmed that liver fibrosis was established after 7 days and animals received BMC transplantation.

### BMC isolation and transplantation

BMC were isolated from the tibias and femurs of two-month old healthy C57BL/6 eGFP^+^ male mice euthanized in a CO_2_ chamber. The medullar canal was exposed and washed with cold DMEM (Dulbecco's Modified Eagle's Medium, Sigma-Aldrich) pH 7.2. After centrifugation at 1500 RPM, BMC were resuspended in red blood cell lysis buffer (10mM NaHCO_3_, 150mM NH_4_Cl, 0.4% EDTA, pH 7.4). After viability test with 5% Trypan Blue, BMC were counted and 1x10^7^ cells were transplanted via jugular vein in mice with 7 days of liver fibrosis.

### Quantitative analysis of collagen in the liver

For quantitative analysis of collagen accumulation, the livers were fixed in 10% formalin and paraffin sections were stained with Picrosirius Red (Sigma Aldrich), which stains collagen fibers, and hematoxylin. To quantify liver fibrosis, 15 random fields per animal, acquired of non-serial sections, were captured in a light microscope with CCD camera. The analysis was made with software Image Pro Plus 3.0 by densitometry of areas stained in red.

### Analysis of hepatic parameters

Blood samples were collected in heparinized tubes and after centrifugation at 2.000 rpm, the serum was obtained. Tests (Bioclin, Brazil) were performed to access the levels of hepatic enzymes GOT (glutamic-oxaloacetic transaminase) and alkaline phosphatase (AP).

### Immunofluorescence to HGF and SDF-1

The tissue was embedded in Tissue Tek and maintained at -70°C until sectioning at 6 μm. Liver sections were then fixed in cold acetone for 10 minutes. To analyze HGF and SDF-1 liver sections were incubated with polyclonal rabbit anti-mouse HGF (Abcam, 83760) or SDF-1 (Abcam, 25117) primary antibody, 1:200 dilution. Then, liver sections were incubated with anti-rabbit Alexa 555-conjugated secondary antibody (Thermo Scientific, A31572), at same proportion of primary antibodies. Sections were mounted with SlowFade (Thermo Scientific). Images were obtained in a Zeiss LSM 510 META confocal laser scanning microscope (Carl Zeiss, Germany).

### Cytometry analyses

#### Immunophenotyping and analysis of cell proliferation in the liver

Frozen tissue was mechanically digested in a solution containing collagenase IA 0.025% (Sigma–Aldrich) and EGTA 0.05mM under agitation in a hotplate for 5 minutes. The cells were collected and washed with cold DMEM supplemented with 1% fetal bovine serum. Then, cells were filtered using Cell Strainer size 70 μm (Corning) and incubated with the following primary antibodies (Table 1): anti-CD144 (endothelial progenitor cells), anti-CD11b (macrophages), anti-CD68 (Kupffer cells), anti-Ly6G (neutrophils) or anti-PCNA (Proliferating cell nuclear antigen) conjugated to phycoerythrin (PE) for 30 minutes at 4°C. After that, cells were washed and resuspended in PBS (phosphate-buffered saline) and analyzed by flow cytometer BD Accuri C6 (BD Biosciences).

**Table 1:** List of antibodies.

Antibody	Catalog Number	Manufacturer
HGF	83760	Abcam
SDF-1 Alexa 555 (Secondary)	25117 A31572	Abcam Thermo Scientific
CD144	138009	BioLegend
CD11b	101207	BioLegend
CD68	137013	BioLegend
Ly6G	108407	BioLegend
PCNA	307908	BioLegend
IL-10, IL-17A, IFN-γ, IL-6, IL-2 and IL-4 (kit)	560485	BD Biosciences

Exclusively to analyze GPF^+^ BMC migration, GFP^+^ BMC proliferation and total proliferation in the first moments after cell therapy, 3 groups were added: mice with 7 days of fibrosis that received BMC transplantation and were euthanized after 3h (F7d+BMC 3h), 24h (F7d+BMC 24h) and 48h (F7d+BMC 48h).

#### Cytokine profiling by cytometric bead array (CBA)

Liver tissue (100 μg) was lysed in lysis buffer (50 mM Tris-HCl pH 7.5, 5 mM EDTA, 150 mM NaCl, 1 mM NaF, 0.2 mM Sodium orthovanadate, 0.1% SDS, 1% Nonidet P40). Cytokines IL-10, IL-17A, IFN-γ, IL-6, IL-2 and IL-4 were measured in liver homogenates using the Kit CBA Mouse Th1/Th2/Th17 (BD Biosciences, described in Table 1) and were analyzed by software FCAP Array 3.0 (BD Biosciences).

### Real time PCR (qPCR)

Total RNA was extracted from 50 mg of macerated liver tissue using Trizol reagent, following manufacturer instructions. RNA amount was determined using Nanovue (GE Life Sciences) spectroscopy, and 1 μg of RNA was treated with DNAse I. Synthesis of the first strand cDNA was performed using Oligo (dT) primers for mRNA and Superscript III reverse-transcriptase. The resulting cDNA (2 μl for each reaction) was then mixed with SYBR Green mix and 0.4 μl from a 10μM of solution of each primer in triplicates. Primers for qPCR were designed using the Primer3 online software and are indicated in Table 2. Reagents were from Thermo Scientific. Quantitative real time PCR (qPCR) was performed using a BioRad CFX96 cycler, and endogenous control GAPDH (Glyceraldehyde 3-phosphate dehydrogenase) was used to normalize the expression of the selected genes. Efficiencies of qPCR for the target gene and the endogenous control were approximately equal, and were calculated through dilution series of cDNA. Real Time PCR reactions were conducted as follows: after a pre-denaturation and polymerase-activation program (4 min at 95°C), forty-four cycles, each one consisting of 95°C for 10 s and 60°C for 15 s were followed by a melting curve program (60 to 95°C with heating rate of 0.1°C/s). Negative controls consisted of wells in which cDNA was substituted for deionized water. The relative expression ratio (RQ) of mRNA was calculated by the equation 2-ΔΔCt, in which -ΔCT expresses the difference between number of cycles (CT) of the target genes and the endogenous control.

**Table 2:** qPCR primers.

Name	5–3’	Primer sequence
HGF	FW	AACTCGGATGTTTGGGTCAG
HGF	RV	CATTCAAGGCCAAGGAGAAG
IL-10	FW	ATGTTGTCCAGCTGGTCCTT
IL-10	RV	TGCTATGCTGCCTGCTCTTA
IL-13	FW	TGGGCTACTTCGATTTTGGT
IL-13	RV	CAGCATGGTATGGAGTGTGG
IFN-γ	FW	TGAGCTCATTGAATGCTTGG
IFN-γ	RV	GGCCATCAGCAACAACATAA
FOXP3	FW	GAGAGGCCTAGAGCCCTGAT
FOXP3	RV	CAGCAGGAGAAAGCGGATAC
GADPH	FW	CCTTCCACAATGCCAAAGTT
GADPH	RV	GGTGCTGAGTATGTCGTGGA

PCR primers were purchased from Thermo Scientific.

### Statistical analysis

Data were analyzed using one-way ANOVA with Tukey’s post-test for multiple comparisons, unpaired t-test (two-tailed), and correlation and linear regression. Data expressing P≤ 0.05 and r^2^ ≥ 0.90 was considered significant. All statistical analysis was performed using GraphPad Prism 5.0 software.

## Results

### Analysis of fibrosis and hepatic parameters

**Fig 1:**
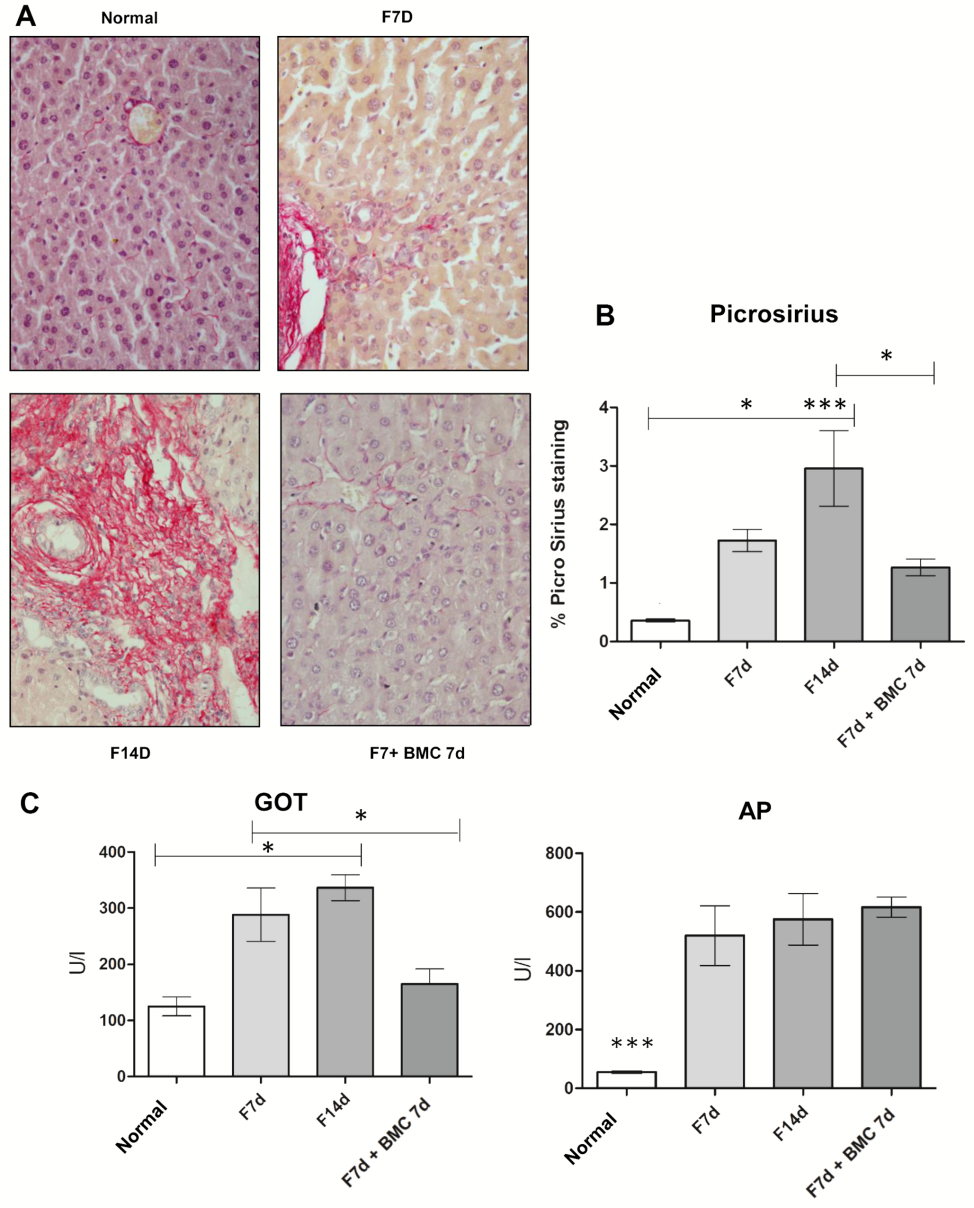
Quantitative analysis of collagen and hepatic parameters. (A) Light microscopy images from Picrosirius staining (40x). (B) Collagen quantification from Picrosirius staining. (C) Analysis of liver enzymes. Cell therapy ameliorated collagen deposition and GOT levels.

Picrosirius staining showed that normal livers presented characteristic distribution of collagen fibers, mainly in portal areas and around centrolobular veins. After 7 and 14 days of BDL, livers presented abnormally increased deposition of collagen in portal areas and proliferation of bile ducts (Fig 1A). These are morphological changes that characterize BDL model. However, after GFP^+^ BMC transplantation, the livers presented preserved parenchyma compared to fibrotic groups and significantly less collagen confirmed by quantification using the software Image Pro Plus 3.0 (Fig 1B and S1 Table).

GOT levels had significant increase in fibrotic groups (7d and 14d) compared to normal animals. However, after GFP^+^ BMC transplantation, the levels of GOT had a significant reduction compared to fibrotic groups. The AP levels had significant increase in fibrosis groups (7d and 14d) compared to normal group, but after GFP^+^ BMC transplantation AP levels were not reduced (Fig 1C and S2 Table).

### Immunofluorescence to SDF-1 and HGF

**Fig 2:**
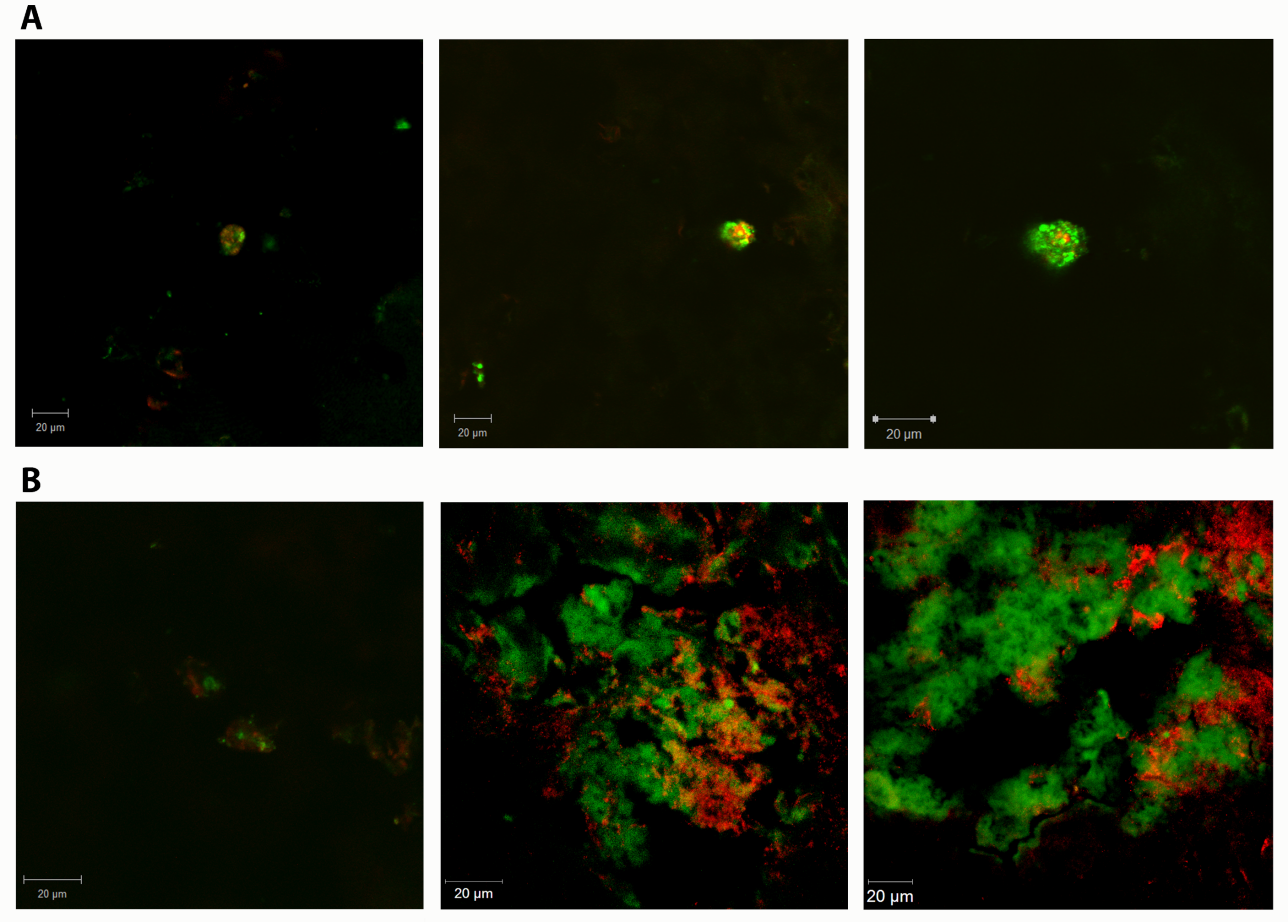
Confocal microscopy images. Immunofluorescence performed in livers from fibrotic animals, obtained 7 days after BMC transplantation, using antibodies to (A) HGF and (B) SDF-1, all in red color. GFP^+^ BMC (green) were found near regions with expression of these proteins in the fibrotic liver. Bar = 20 μm.

Immunofluorescence images obtained from F7d+BMC 7d group using antibody anti-HGF (Fig 2A) showed diffuse staining for this protein in the liver parenchyma, and GFP^+^ BMC were found near regions expressing this factor in the fibrotic liver. SDF-1 expression was prominent in scattered areas of the liver parenchyma, and GFP^+^ BMC were commonly near SDF-1 positive areas (Fig 2B).

### Cytometry analyses

All cytometry data within this study is available in S3 Table.

#### Cytometric analysis of total liver populations: GFP^+^ BMC migration to the liver, cell proliferation analysis and immunophenotyping

**Fig 3:**
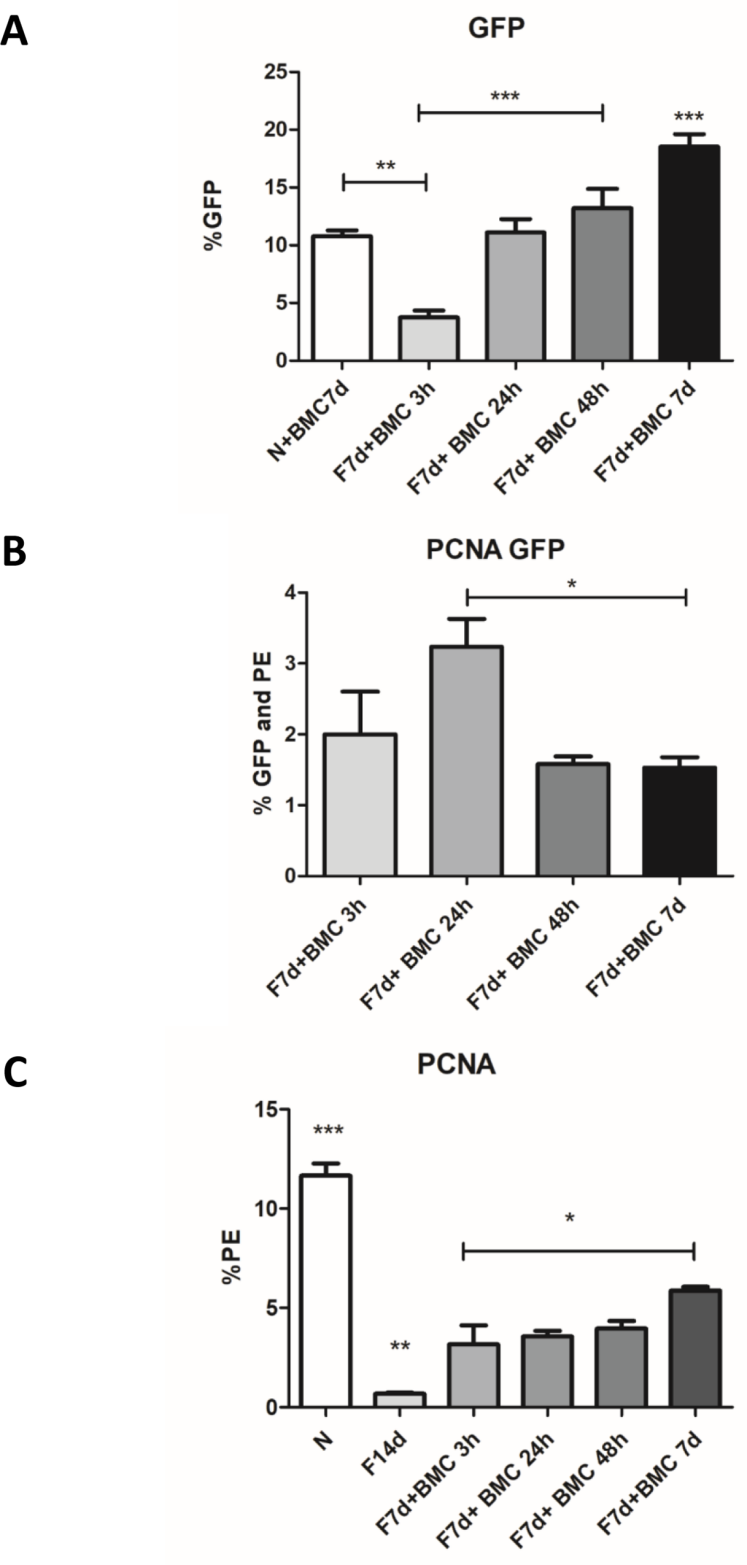
GFP^+^ BMC migration and cellular proliferation in fibrotic liver. (A) Percentage of GFP^+^ BMC cells in the liver (% GFP). (B) GFP^+^ BMC proliferation, showing percentage of GFP^+^ BMC with PCNA Staining (% GFP and PE) per all GFP^+^ cells. (C) Total cell proliferation in the liver (% PE) ***P = <0.0001, **P = <0.001 and *P<0.05.

Flow cytometry analysis of liver dissociated cells showed that GFP^+^ BMC homing to the liver was increased in fibrotic group after 7 days of BMC transplantation (18.54% of all liver cells) compared to transplanted normal group (10.76%) and earlier transplanted fibrotic groups: F7d+BMC 3h (3.73%), F7d+BMC 24h (11.08%) and F7d+BMC 48h (13.20%). Therefore, the increase in the number of established BMC cells in the liver of transplanted fibrotic groups was time-dependent until the time-point studied (Fig 3A).

Regarding GFP^+^ BMC proliferation (GFP^+^ PCNA^+^ cells), there was a significant increase in proliferation 24 hours after transplantation (3.23% of all GFP^+^ cells were PCNA^+^) compared to 48h (1.91%) and 7 days (1.53%) (Fig 3B).

Total liver cell proliferation analysis using PCNA marker showed a significant reduction in 14d fibrotic group (where 0.71% of all liver cells were PCNA^+^) compared to the normal group (11.45%), corroborating extensive and widespread cell damage caused by fibrosis at this stage of consolidated disease. However, the transplanted groups: F7d+BMC 3h (3.15%), F7d+BMC 24h (3.55%), F7d+BMC 48h (3.95%) and F7d+BMC 7d (5.86%) had significant increase in total cell proliferation compared to non-transplanted fibrotic group. F7d+BMC 7d showed significant increase compared to the other transplanted groups (Fig 3C).

**Fig 4:**
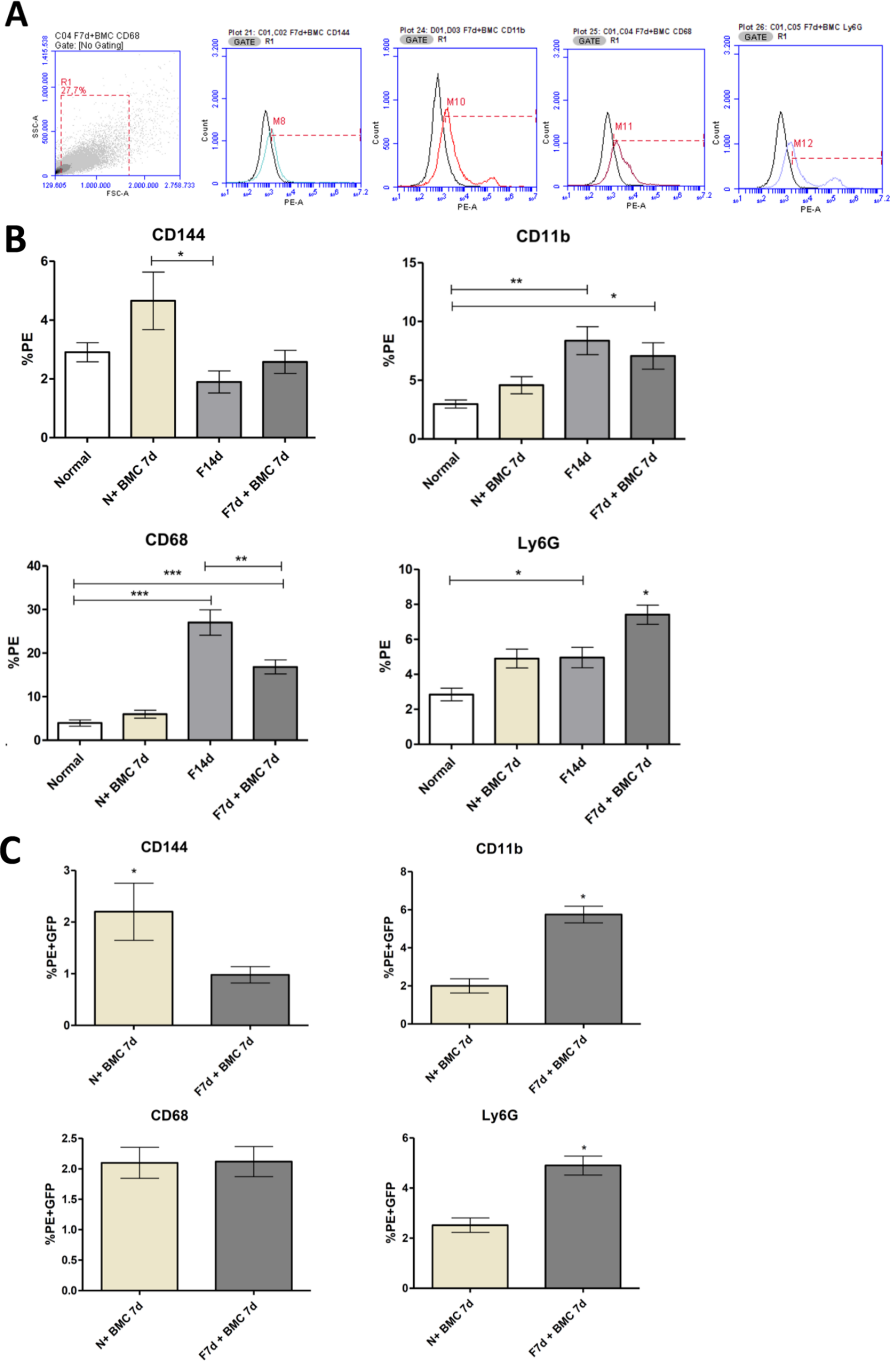
Characterization of total liver cells and transplanted GFP^+^ BMC. (A) Forward scatter x side scatter graph showing typical morphological measurements of hepatic cells after enzymatic dissociation. The histograms show unstained cells (black curves) and stained cells to CD144, CD11b, CD68 and Ly6G antibodies (colorful curves). Quadrant chart shows double marking selection. (B) Immunophenotyping of total hepatic cells populations: CD144, CD68, CD11b and Ly6G. (C) Analysis of transplanted GFP^+^ BMC in the liver, described as the percentage of liver dissociated cells that emitted GFP fluorescence and marked to the antibodies CD144, CD68, CD11b and Ly6G. ***P = <0.0001, **P = <0.001 and *P<0.05.

Liver dissociated cells were also studied to identify the prevalence of selected subpopulations in each experimental group (**Fig 4B**). Regarding the total percentage of CD144 cells (endothelial progenitor cells), there was a significant increase in the normal transplanted group (5.96%) compared to the fibrotic group (F14d, 1.89%). Expression of CD68 (Kupffer cells) showed a significant increase in fibrotic group (27.01%) compared with the normal group (3.97%) and normal transplanted group (5.98%) However, the transplanted fibrotic group showed a significant reduction in the percentage of CD68 cells (16.83%). To CD11b expression (extrahepatic macrophages), a significant increase in fibrotic group (8.37%) compared to the normal group (2.97%) was confirmed. Although the transplanted fibrotic group had a mild decrease (7.06%), this was not significant. Ly6G expression (neutrophils) had a significant increase in fibrotic group (4.96%) when compared to normal group (2.85%). The transplanted fibrotic group (7.41%) had a significant increase in Ly6G in comparison to all groups.

#### Cytometric analysis of cell subpopulations derived from transplanted GFP^+^ BMC in the liver parenchyma

The possible differentiation and identity of transplanted GFP^+^ bone marrow cells that migrated and established in liver was assessed using the same antibodies described above for characterization of hepatic subpopulations. In relation to these transplanted cells (cells positive for both PE and GFP), the transplanted normal group showed a significantly higher percentage of CD144 positive cells (endothelial progenitor cells) after transplantation (2.2 ± 0.55) compared to the transplanted fibrotic group (0.88 ± 0.09). Meanwhile, the percentage of GFP cells positive to CD11b (extrahepatic macrophages) or Ly6G (neutrophils) was significantly higher in transplanted fibrotic group (5.75 ± 0.44 and 4.90 ± 0.37 respectively) compared to the transplanted normal group (2.0 ± 0.37 and 2.52 ± 0.28). There was no significant difference in the percentage of Kupffer cells between both groups (Fig 4C).

**Fig 5:**
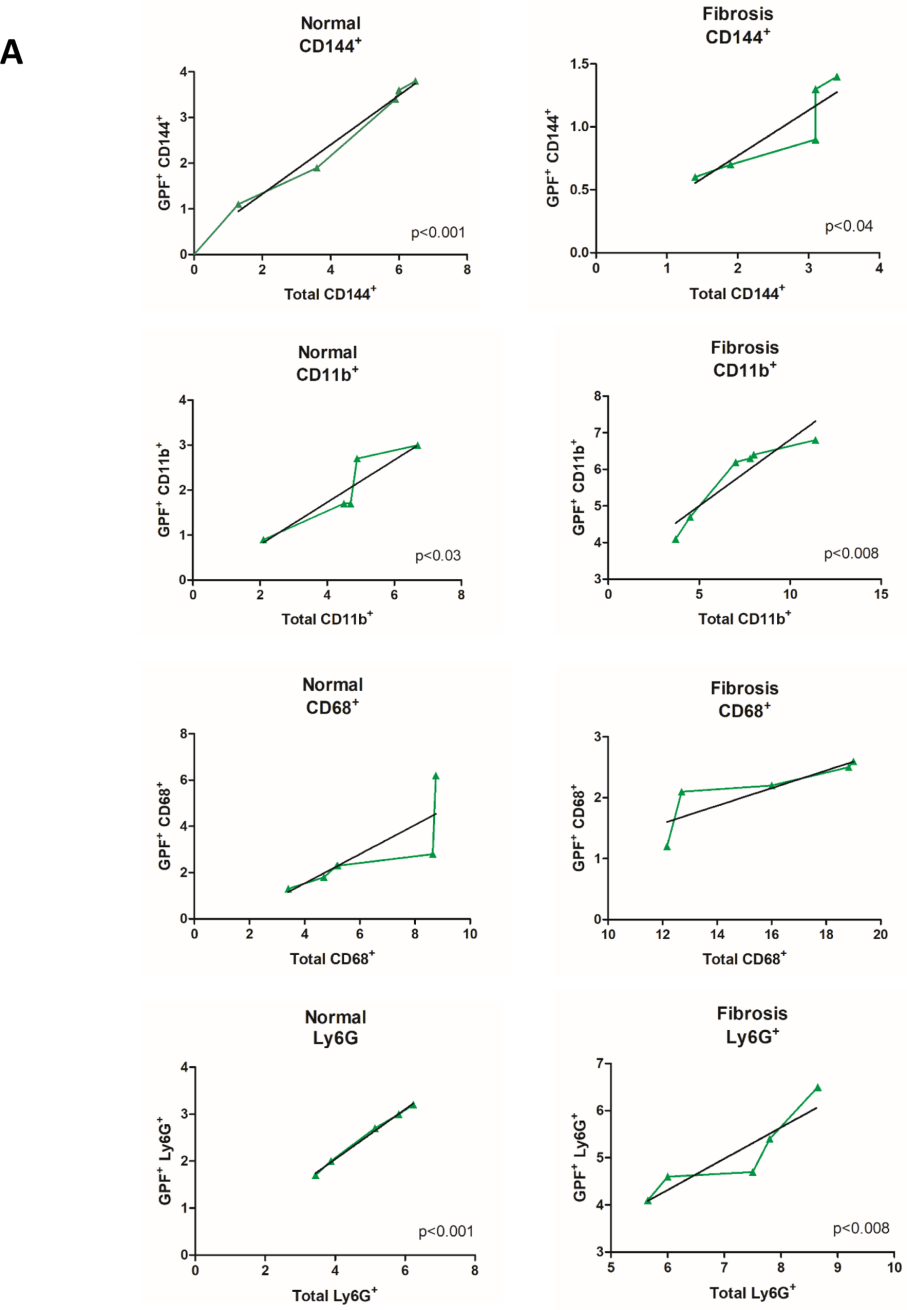
GFP^+^ BMC correlation tests. (A) Correlation between GFP^+^ BMC marked with PE and total cell populations marked with CD144, CD68, CD11b and Ly6G. Positive correlation for CD144, CD11b and Ly6G in transplanted normal and fibrotic groups (N+BMC7d and F7d+BMC7d).

Correlation tests between the percentages of GFP^+^ BMC that migrated to the liver and the total percentage for each cell population analyzed showed positive results to populations of endothelial progenitor cells, extrahepatic macrophages and neutrophils in transplanted groups (normal and fibrosis). These data show that there was a significant contribution of GFP^+^ BMC to the total populations of these cells in the liver. There was no correlation with the percentage of Kupffer cells in both groups (Fig 5A).

#### Cytokine profiling by cytometric bead array (CBA)

**Fig 6:**
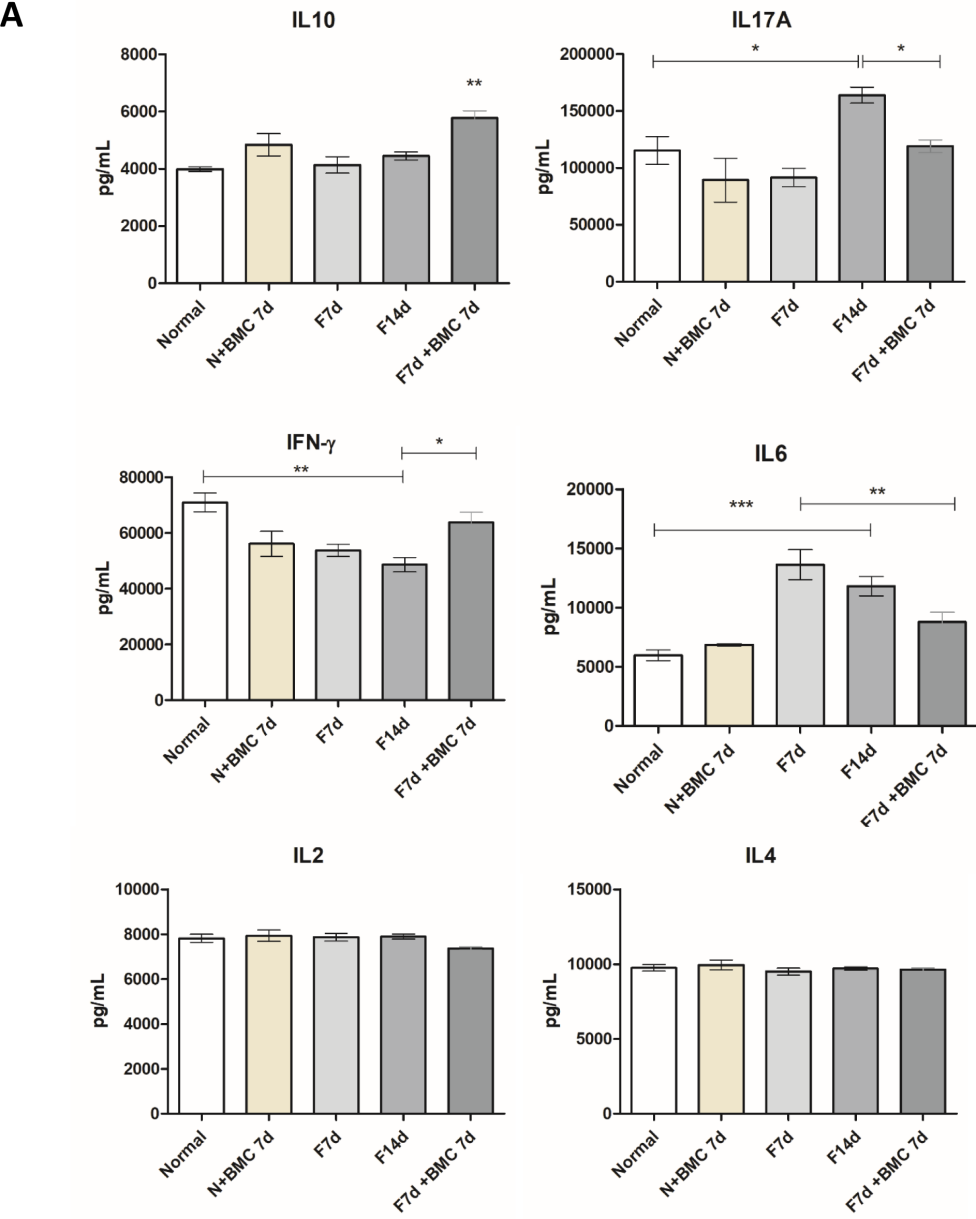
Cytokine profiling. IL-10, IL-17A, IFN-γ, IL-6, IL-4 and IL-2 protein content was analyzed using a flow cytometry-adapted cytokine kit. ***P = <0.0001, **P = <0.001 and *P<0.05.

Cytokine IL-10 presented a significant increase in transplanted fibrotic group compared to the other groups. For IL-17A, there was a significant increase in F14d group compared to the normal group, and after BMC transplantation there was a significant reduction. For IFN-γ, F7d, F14d and transplanted normal groups had significant reduction compared to the normal group. However, the transplanted fibrotic group showed a significant increase, similar to the normal group. For cytokine IL-6, F7d and F14d groups showed significant increase compared to the normal group and after BMC transplantation there was significant reduction compared only to F7d. There were no significant differences between the groups to the cytokines IL-4 and IL-2 (Fig 6).

### Real time PCR (qPCR)

**Fig 7:**
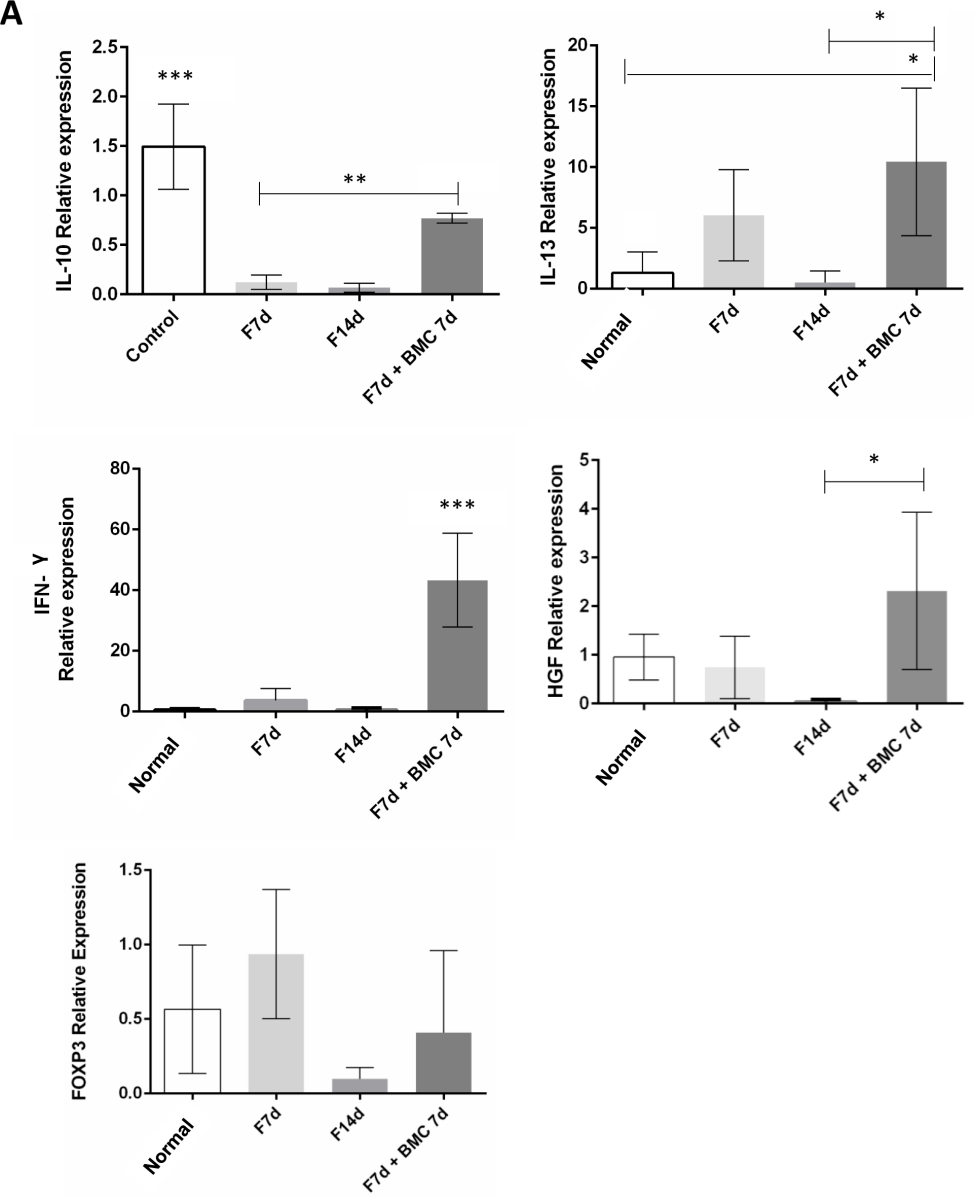
Cytokines gene expression. IL-10, IL-13, IFN-γ, HGF and FOXP3 genes expression was measured through qPCR. ***P = <0.0001, **P = <0.001 and *P<0.05.

Gene expression analysis showed that, as well as observed in CBA protein analysis, IL-10 expression was significantly increased in the transplanted fibrotic group compared to fibrotic groups, corroborating that cytokine as an important factor for fibrogenic cells apoptosis in hepatic chronic injuries. The anti-inflammatory cytokine IL-13 expression was also significantly increased in the transplanted fibrotic group. IFN-γ expression was highly increased in the transplanted fibrotic group, as in liver fibrosis this cytokine plays an anti-fibrotic and pro-ECM remodeling role. HGF, an important survival and proliferation factor in the liver, had its expression decreased in fibrotic groups and increased after BMC therapy. However, FOXP3 (forkhead box P3) expression, an important transcription factor to regulatory T (Treg) cells, was not changed with BMC transplantation (Fig 7 and S4 Table).

## Discussion

Although the pathogenesis of liver fibrosis is well characterized in diverse types of hepatic diseases, there is little data on cell and molecule interplay in the regenerating hepatic microenvironment after cell therapy. Nevertheless, this work showed for the first time the phenotypic cell profile of transplanted bone marrow cells that migrated and established in the fibrotic liver with cholestatic disease, while correlating these results with cytokine profile expression.

In this work, BMC transplantation induced normalization of GOT enzyme, and reduction in collagen deposition in the fibrotic liver, corroborating our previous results [17]. We also demonstrated that there was differential migration of BMC to the liver depending on preexisting conditions of the organ. The percentage of BMC that established in healthy liver after 7 days is nearly 10.76%. The liver is between the organs that have the highest rates of transplanted stem cells migration under physiological conditions [18]. The percentage of BMC that established in fibrotic liver was significantly higher, an average of 18.54%. During an injury, parenchymal and inflammatory cells release different chemoattractants that influence on BMC migration, which may explain the increase of these cells in liver fibrosis [19]. Our results showed that within the first 48 hours after transplantation a large number of GFP^+^ BMC proliferated and established in the hepatic parenchyma. However, among the 2^nd^ and 7^th^ day after transplantation, BMC number barely increased by around 4%.

Confocal microscopy analysis showed transplanted BMC in regions near SDF-1 and HGF expression. SDF-1 is acknowledged as a leukocyte chemoattractant, and its receptor CXCR4 is present on hematopoietic stem cells and in a small proportion of mesenchymal stem cells, where it is responsible to mobilize the migration of these cells from the bone marrow out to the blood flow. During fibrosis, we observed that in the transplanted group, BMC were most frequently found near SDF-1 expressing sites in the hepatic parenchyma. Studies show that in chronic liver disease, proinflammatory cytokines such as TNF-α, IL-1β and PDGF (platelet-derived growth factor) stimulate the secretion of SDF-1 by hepatocytes in the tissue [20,21]. Our results strongly suggest that SDF-1 may act as a BMC chemoattractant in the model of liver fibrosis with cholestatic disease.

Similar results were observed regarding HGF expression in our model. Although this cytokine is mainly expressed by hepatocytes, recent data showed that mesenchymal stem cells found in bone marrow could also secrete HGF. In fact, qPCR analysis showed that HGF gene expression increased after BMC transplantation. Besides stimulating hepatocyte proliferation, HGF plays an anti-fibrotic action by inducing apoptosis of fibrogenic cells such as activated HS and myofibroblasts [15,22,23]. Altogether, confocal microscopy and qPCR results showed that HGF acts as a chemotactic factor of BMC while its increased expression in liver is indicative of hepatic regeneration.

Total cell proliferation analysis showed that healthy liver displays a considerable level of proliferation, due to physiological turnover of hepatocytes. However, during cholestatic fibrosis there was a significant reduction of proliferating cells in the liver, compromising its homeostasis. Previous results from the group showed that in early fibrosis, increased cell proliferation related to higher amounts of fibrogenic cells, and only when fibrosis is well established, PCNA levels decreased. However, after BMC transplantation, parenchymal cell proliferation was increased while only fibrogenic cells suffered apoptosis, demonstrating the activation of a mechanism of hepatic tissue remodeling [24]. Moreover, in this work we showed that among all proliferating cells, BMC correspond to a small portion of total proliferating cells. BMC transplantation associated with increased proliferation of overall liver cell populations, when compared to the non-transplanted fibrotic group, suggesting possible hepatocyte proliferation and renewal of liver parenchyma induced by BMC.

Characterization of hepatic populations showed that BMC transplantation significantly changed cell populations in the liver of transplanted groups. In relation to endothelial progenitor cells analysis, the transplanted normal group presented a significant increase, and correlation test showed that some of these cells were directly BMC-derived. Furthermore, the percentage of these cells in transplanted fibrotic group was similar to the normal group, and a small number was BMC-derived. Endothelial progenitor cells have high regenerative potential and secrete angiogenic factors, such as VEGF (vascular endothelial growth factor) and HGF. Even a small increase in the number of endothelial cells during fibrosis, as observed in our results after cell therapy, could have an important role in tissue remodeling. However, these cells are also associated with tissue tumorigenesis and metastasis, and it is noteworthy that only when healthy animals were injected with BMC, endothelial progenitor cells percentage was highly increased, suggesting that the safety of BMC transplantation is associated with the presence of a chronic liver injury in our model [25,26].

Regarding Kupffer cells, there was a significant increase in the fibrotic group compared to normal group, but after BMC transplantation, these cells significantly reduced in the liver. There was no correlation between Kupffer cells population and the transplanted GFP^+^ BMC. During chronic injury, activated Kupffer cells secrete pro-inflammatory cytokines and chemoattractants, followed by a high expansion in hepatic macrophages, and recruitment of immune cells [27,28].

There was a significant increase in extra hepatic macrophages in the fibrotic group compared to the normal group. Despite the percentage in transplanted fibrotic group being less than the fibrotic group, there was no significant difference. However, within that percentage of total extra-hepatic macrophages, 5.75% were BMC-derived in the transplanted fibrotic group. Depending on the stimulus, macrophages can be polarized in M1, with pro-inflammatory profile and release of cytokines such TNF-α, IL-1β, IL-12 and reactive oxygen species (ROS) or M2, anti-inflammatory and immuno-suppressive profile with release of cytokines such as IL-10, IL-14 and IL-13 [29,30,31]. Extrahepatic macrophages derived from circulating monocytes are attracted to injured sites and extend the damage with pro-inflammatory and pro-fibrotic actions (profile M1) as seen in the fibrotic groups through cytokine profiling. However, macrophages derived from BMC seem to represent a different population, and are capable of reducing hepatic fibrosis through the production of MMPs, mainly MMP-9 and MMP-13 as previously described by our group [14]. Furthermore, our present results add new evidence to this hypothesis, showing that after BMC transplantation, anti-inflammatory cytokines IL-10 and IL-13 levels are increased.

IL-13 is an immunosuppressive cytokine that stimulates macrophages polarization (M2 profile) and consequently IL-10 expression [13, 32]. In the present work, we observed that transplanted BMC were responsible by macrophage population increase in liver, followed by IL-10 and IL-13 increase. These data indicate an anti-inflammatory immunomodulation and suggest possible exchange in macrophages to M2 profile with cell therapy. We hypothesize that monocyte precursors from the transplanted BMC are able to rebalance the inflammatory microenvironment. Once these cells do not suffer the early inflammatory events that occur soon after fibrosis is induced, which in our model are caused by bile acids accumulation in the liver, they are preserved from the initial inflammatory adverse effects and directed towards a M2 profile, that favors tissue remodeling and regeneration.

The analysis of neutrophils showed a significant increase in the fibrotic group compared to the normal group, the higher number of neutrophils being found in transplanted fibrotic group. In rats, BDL model demonstrated that even when depleted of neutrophils, there were no differences in hepatic fibrogenesis compared to control animals. It has been demonstrated that neutrophils can contribute to decrease collagen during resolution of fibrosis, through the production of MMPs -9 and -13 [33,34,14]

Cytokine measurements demonstrated an increase of pro-inflammatory and fibrotic interleukins such as IL-6 and IL-17A in F14d group. However, these cytokines had reduced levels after BMC transplantation. IL-6 is a known pro-inflammatory cytokine that in the presence of increased levels of TGF-β, as in hepatic fibrosis, promotes the differentiation of naiive T lymphocytes to Th17 cells, characterized by autoimmunity and secretion of a pro-inflammatory cytokine profile, including IL-17A. Multiple cells express IL-17 receptor, such as monocytes, Kupffer cells, HSC and colangiocytes, that in response to activation by IL-17, amplify the inflammatory process through secretion of IL-1β, IL-6, TNF-α and TGF-β. Cytokine IL-17 also plays an important role in the migration of pro-inflammatory neutrophils to the injured liver. Therefore, decrease of these two cytokines, observed after BMC transplantation in our model indicates an anti-inflammatory action recognized as essential to reverse liver fibrosis [8,35,36].

Cytokine IFN-γ plays an anti-fibrotic role in the liver because it inhibits the activation of HS and ECM production through downregulation of transcriptional genes that encode TGF-β, PDGF and TNF-α. Recently it has been proposed that the increase of IFN-γ expression in the fibrotic liver is an important factor that halts the progression of fibrosis. IFN-γ can be produced by natural killer (NK) cells, T CD8 and Th1 cells. Although NK cells are responsible for hepatocyte apoptosis via TNF-α TRAIL ligand, these cells have drawn attention because in certain circumstances, they act as inhibitors of hepatic fibrosis. NK cells from patients with liver fibrosis caused by hepatitis were able to promote activated HS apoptosis. Our analysis showed that IFN-γ expression was highly increased in the transplanted fibrotic group, indicating that an important antifibrotic response occurs after BMC transplantation [34,37,38].

Gene expression analysis showed that FOXP3 (forkhead box P3), an important transcription factor to Treg cells, was not changed with BMC transplantation, suggesting other possible pathways for anti-inflammatory modulation through cell therapy in the liver. Indeed, Treg cells may turn into Th17 cells under certain microenvironmental conditions such as in IL-6 and TGF-β presence, and produce proinflammatory factors, which could halt liver regeneration [39].

## Conclusion

In summary, BMC participation in liver regeneration from chronic injury follows a complex and coordinated mechanism of immune regulation mediated by different phenotypes from these cells, resulting in tissue remodeling and restoration of liver function.

## Supporting information

S1 Table Picro sirius measurements. Raw data regarding Picro Sirius staining quantification by densitometry; numbers indicate the percentage of stained area over total area of the captured microscopy field.
(XLSX)

S2 Table Liver enzymes measurements. Raw data regarding liver enzymes after analysis by spectrophotometry.
(XLSX)

S3 Table Flow cytometry data. Raw data regarding each cytometric analysis; numbers indicate the percentage of each population analyzed over total cells or populations.
(XLSX)

S4 Table Quantitative PCR data. Raw data regarding PCR analysis. The equation 2-ΔΔCt was applied to obtain the relative expression ratio (RQ) of mRNA. Each -ΔCT expresses the difference between number of cycles (CT) of the target genes and the endogenous control.
(XLSX)

## Abbreviations

AP: alkaline phosphatase
BDL: bile duct ligation
BMC: bone marrow cells
DMEM: Dulbecco's modified Eagle's medium
ECM: extracellular matrix
FOXP3: forkhead box P3
GAPDH: Glyceraldehyde 3-phosphate dehydrogenase
GOT: glutamic-oxaloacetic transaminase
HGF: hepatocyte growth factor
HS: hepatic stellate cells
HSC: hematopoietic stem cells
MMP: metalloproteinases
MSC: mesenchymal stem cells
NK: natural killer
PCNA: proliferating cell nuclear antigen
PDGF: platelet-derived growth factor
ROS: reactive oxygen species
SDF-1: stromal derived factor-1
TGF-β: transforming growth factor beta
TNF-α: tumor necrosis factor-alpha
